# Hydrogen gas promotes the adventitious rooting in cucumber under cadmium stress

**DOI:** 10.1371/journal.pone.0212639

**Published:** 2019-02-20

**Authors:** Bo Wang, Biting Bian, Chunlei Wang, Changxia Li, Hua Fang, Jing Zhang, Dengjing Huang, Jianqiang Huo, Weibiao Liao

**Affiliations:** College of Horticulture, Gansu Agricultural University, Yinmen Village, Anning District, Lanzhou, PR China; Hainan University, CHINA

## Abstract

Hydrogen gas (H_2_) plays an important role in plant development and stress responses. Here, cucumber (*Cucumis sativus* L.) explants were used to investigate the roles of H_2_ in adventitious root development under cadmium (Cd) stress and its physiological mechanism. The results showed that hydrogen-rich water (HRW) promoted adventitious rooting under Cd stress and 50% HRW obtained the maximal biological response. Compared with Cd treatment, HRW + Cd treatment significantly reduced the content of malondialdehyde (MDA), hydrogen peroxide (H_2_O_2_), superoxide radical (O_2_^-^), thiobarbituric acid reactive substances (TBARS), ascorbic acid (AsA) and reduced glutathione (GSH), as well as relative electrical conductivity (REC), lipoxygenase (LOX) activity, AsA/docosahexaenoic acid (DHA) ratio, and GSH/oxidized glutathione (GSSG) ratio, while increasing DHA and GSSG content. HRW + Cd treatment also significantly increased in the activity and related gene expression of ascorbate peroxidase (APX), dehydroascorbate reductase (DHAR), monodehydroascorbate reductase (MDHAR) and glutathione reductase (GR). Additionally, HRW + Cd treatment increased the contents of osmotic adjustment substances, as well as the activities of peroxidase (POD) and polyphenol oxidase (PPO), while significantly decreasing indoleacetic acid oxidase (IAAO) activity. In summary, H_2_ could induce adventitious rooting under Cd stress by decreasing the oxidative damage, increasing osmotic adjustment substance content and regulating rooting-related enzyme activity.

## Introduction

Cadmium (Cd) is one of the toxic heavy metals, which has become a major pollutant caused by use of agrochemicals, industrial waste and mining activities. Cd can be easily absorbed by plants and enter the human body by the consumption of Cd-containing food, posing a serious risk to human health [1]. The visible symptoms of Cd toxicity in plant include chlorosis, programmed cell death (PCD) and even cell death [2]. Cd stress also results in the inhibition of plant growth, transpiration, photosynthesis, respiratory, nitrogen and protein metabolisms [3]. Cd could alter the uptake of minerals by plants [4]. Excess Cd also severely destroyed plant structure [5]. Excess Cd could destroy antioxidant defense system in plants, reducing the activities of antioxidant enzymes and the levels of non-enzymatic antioxidant, which caused the over accumulation of lipid peroxidation and reactive oxygen species (ROS) in plants [6–7]. As we know, antioxidant enzymes can act as detoxifiers to maintain a balance between ROS production and elimination [8].

Hydrogen gas (H_2_), a colorless, tasteless diatomic gas, is a novel and effective antioxidant in plants and animals [9]. It is documented that H_2_ is an effective anti-oxidative and anti-inflammatory agent with important medical value. However, direct use of H_2_ is dangerous and flammable. Therefore, most of researchers use the hydrogen-rich water (HRW) to perform experiments, which is safe, cost-effective and commercially available. Recently studies found that H_2_ is a potent physiological regulatory agent for animals [10]. In plants, previous studies reported that H_2_ might be produced in several species [11] and promoted the seed germination in winter rye [12]. Recent studies have revealed that H_2_ could enhance plant growth and development [13]. For example, H_2_ had positive effects on plant growth in mung bean [14] and postharvest senescence in kiwifruit by decreasing the lipid perxidation level [15]. In addition, it was reported that H_2_ could regulate stomatal closure [16] and root development in plants [17]. H_2_ was also an effective anti-stress molecule for plants in abiotic stress adaptability, such as the tolerances of salt, mercury (Hg), Cd, aluminum, heat and high light [9, 18, 19]. H_2_ could regulate the expression of responsive genes during adventitious root development [20] and anthocyanin biosynthesis [21]. More importantly, H_2_ was an antioxidant that could selectively reduce cytotoxic free radicals [22].

Cucumber (*Cucumis sativus* L.) is widely planted in China, and its productivity and quality greatly reduced due to abiotic stresses, such as Cd, salt and drought stresses. As mentioned above, H_2_ could alleviate Cd toxicity to plant growth and development. Therefore, here, we investigated the physiological and biochemical response in cucumber during H_2_-induced adventitious rooting under Cd stress.

## Materials and methods

### Plant material and growth conditions

Selected identical seeds of cucumber (*C*. *sativus* ‘Xinchun 4’; Gansu Academy of Agricultural Sciences, Lanzhou, China) were soaked in distilled water for 5 h. The seeds were germinated on filter paper with distilled water in petri dishes and maintained at 25 ± 1°C for 5 days with a 14-h photoperiod at 200 μmol m^-2^s^-1^ intensity in an illuminating incubator. The 5-d-old cucumber seedlings whose primary roots were removed were used as explants. The explants were placed in petri dishes with distilled water or different chemicals indicated below under the same conditions of temperature and photoperiod described above for another 5 days. The number of adventitious roots per explants was observed and recorded, and corresponding photographs were taken.

### Explants treatments

Cucumber explants were placed in the culture dish with 50 mL distilled water (control) or various concentrations of Cd(NO_3_)_2_ solution (0.25, 0.5, 1, 2 and 4 μM), and then incubated in an illuminating incubator for 5 days. The concentration of moderate Cd stress was selected for subsequent experiments according to the rooting index. The following chemicals were added alone or together with Cd(NO_3_)_2_: (1) distilled water (Control); (2) 1 μM Cd (NO_3_)_2_; (3) 1% HRW + 1 μM Cd(NO_3_)_2_; (4) 10% HRW + 1μM Cd(NO_3_)_2_; (5) 50% HRW + 1 μM Cd(NO_3_)_2_; (6) 100% HRW+1 μM Cd(NO_3_)_2_. After 5 days of treatment, the concentration of HRW was selected based on the results of a preliminary experiment in our laboratory. The treatments were arranged in a completely randomized design with at least three replications. In the following experiment, we set four treatments to determine the roles of HRW in adventitious rooting in cucumber under cadmium stress. The treatments are as follows: distilled water (control); 50%HRW; 1 μM Cd(NO_3_)_2_; 50%HRW + 1 μM Cd (NO_3_)_2_. All solutions were changed every 24 hours. The samples were taken at 0, 12, 24 and 48 h and stored in an ultra-low temperature freezer at -80°C [23].

### Preparation of HRW

Purified H_2_ gas (99.99%, v/v) generated from a hydrogen gas generator (QL-300, Saikesaisi Hydrogen Energy Co., Ltd, Shandong, China) was bubbled into 500 mL distilled water at a rate of 330 mL min^-1^ for 15 min. Then, the corresponding HRW was rapidly diluted to the required saturations [1%, 10%, 50% and 100% (v/v)]. H_2_ concentration in freshly prepared HRW was determined by gas chromatography and it remained at a relative constant level in 25°C for at least for 12 h.

### Membrane lipid peroxidation determination

Determination of malondialdehyde (MDA) content: the MDA content in plants was determined by thiobarbituric acid method. Briefly, cucumber explants (0.5 g) were homogenized in a mortar with10 mL of trichloroacetic acid. The mixture was heated at 95°C for 15 min, and then quickly cooled in an ice bath. After centrifuged at 1699 × *g* for 10 min, the absorbance of the supernatant was recorded at 450, 532 and 600 nm, respectively.

Measurement of relative electrical conductivity (REC): cucumber explants (0.1 g) at different treatment times were placed into test tubes (10 mL water) and soaked overnight at room temperature. The measured result on the next day as R1, then the soaking solution was heated with boiling water bath for 30 minutes and cooled. The next measured conductivity of the soaking solution as R2. Relative conductivity = R1/R2 * 100%.

Lipoxygenase (LOX) activity measurement: LOX activity was analyzed as described previously according to the method of Zhang et al [24].

### Measurements of reactive oxygen species

Determination of hydrongen peroxide (H_2_O_2_) content: cucumber explants (0.2 g) were homogenized in an ice bath with 2 mL of 0.1% (w/v) TCA. The homogenate was centrifuged at 1699 × *g* for 10 min and 0.5 mL of the supernatant was added to 0.5 mL of 10 mM potassium phosphate buffer (pH 7.0) and 1 mL of 1 M KI. The H_2_O_2_ content was estimated by measuring the spectrum absorbance of the supernatant at 415 nm and using a standard curve plotted with a known concentration of H_2_O_2_.

Determination of determine superoxide radical (O_2_^-^) content: O_2_^-^ was measured by monitoring nitrite formation from hydro-xylamine in the presence of O_2_^-^, according to the method of Jabs et al [25] with some modifications. Cucumber explants (1 g) at different treatment times were homogenized in an ice bath with 3 mL of 65 mM potassium phosphate buffer (pH 7.8) and centrifuged the mixture at 5000 × *g* for 10 min. The incubation mixture contained 0.9 mL of 65 mM phosphate buffer (pH 7.8), 0.1 mL of 10 mM hydroxylamine hydrochloride, and 1 mL of the supernatant. After incubation at 25°C for 20 min, 17 mM sulfanilamide and 7 mM naphthylamine were added to the incubation mixture. After reaction at 25°C for 20 min, the same volume of ethyl ether was added and the mixture was centrifuged at 1500 × *g* for 5 min. The absorbance in the aqueous solution was recorded at 530 nm. The content of O_2_^-^ was estimated by measuring the spectrum absorbance of the supernatant at 530 nm and using a standard curve plotted with a known concentration of NO_2_^-^.

Diaminobenzidine (DAB) staining analysis: the DAB staining solution (0.1 mg mL^-1^) was dissolved in 50 mM Tris-acetic acid (pH 5.0). Leaves were placed in the dye solution overnight. The next day, leaves were soaked in a fixing solution (ethanol: lactic acid: glycerol = 3:1:1), boiled for 10 min, and then cooled. Under the catalysis of peroxidase, DAB and H_2_O_2_ can generate brown compounds, which can locate hydrogen peroxide in tissues.

Staining of nitroblue tetrazolium (NBT): stress-induced generation of O_2_^-^ in situ was detected by NBT staining [26].leaves were immersed with 0.1% solution of NBT in 10 mM potassium phosphate buffer (pH 7.8) with 10 mM sodium azide NaN_3_, and then incubated in the darkness at 22°C for 10 min until a purple-blue color became visible.

Determination of thiobarbituric acid reactive substances (TBARS) content: lipid peroxidation was estimated by measuring the amount of TBARS according to the method of Cui et al [27].

### Determination of ascorbate-glutathione cycle

Determination of ascorbic acid (AsA) and docosahexaenoic acid (DHA): samples were taken at different treatment times, ground, and centrifuged. The supernatant was used for the AsA assays. The mixture of 0.2 mL of supernatant, 0.5 mL of phosphate buffer (pH 7.4) and 0.1 mL of 10 mM dithiothreitol (DTT) was used to stand at 40°C for 60 minutes. The other steps of DHA are similar to those described above, except that H_2_O instead of DTT and N-ethylmaleimide. The absorbance was measured at 525 nm.

Determination of reduced glutathione (GSH) and oxidized glutathione (GSSG): Samples were taken at different treatment times, ground, and centrifuged. The supernatant was used for GSH and GSSG determinations. The reaction mixture contained 0.2 mL supernatant, 0.05 mL H_2_O, 0.5 mL of 2.5 mM ethylenediaminetetraacetic acid, 0.1 mL of 0.5 mM nicotinamide adenine dinucleotide phosphate, 0.1 mL of 6 mM 2-nitrobenzoic acid and 0.05 mL GR (10 UmL^-1^). The absorbance was measured at 412 nm and GSSG was reduced by GR and then calculated.

Assay of ascorbate-glutathione cycle related enzymes activity: glutathione reductase (GR) activity was analyzed according to previous methods [28]. Ascorbate peroxidase (APX) activity was measured by the methods described previously [29]. The activity of monodehydroascorbate reductase (MDHAR) and dehydroascorbate reductase (DHAR) was measured according to the methods previously reported [30].

### Assay of osmotic adjustment substances content

Proline (Pro) was measured as previously described [31]. Soluble sugar was determined according to the method of Buysse and Merckx [32]. The soluble protein content was measured following the method of Bradford [33].

### Rooting related enzyme assays

Cucumber explants were measured at 4 h after treatment to determine these enzyme activities. For the enzyme extraction, 0.5 g fresh cucumber explants were homogenized in 0.05 M potassium phosphate buffer containing 1% polyvinylpyrrolidone (v/v).

Determination of peroxidase (POD) activity: 0.1 mL enzyme extract was added to substrate mixture containing 1 mL of 0.05 M potassium phosphate buffer, 9 mL of 0.05 M guaiacol (pH 5.5) and 21 mL of 2% H_2_O_2_. Then the reaction was stopped by adding 2 mL of 20% trichloroacetic acid in an ice bath. Then the OD was monitored at 420 nm.

Determination of polyphenol oxidase (PPO) activity: 0.5 mL enzyme solution was added to substrate mixture, which contains 1 mL of 0.1 M catechol and 3.9 mL of 0.05 M phosphate buffer (pH 5.5). The reaction was terminated by adding a certain drug in an ice bath, and then the OD was monitored at 525 nm.

Determination of indoleacetic acid oxidase (IAAO) activity: the substrate mixture [1 mL enzyme extracts (1 mL PBS instead of enzyme solution was added to control), 1 mL 2,4-dichlorophenol, 1 mL MnCl_2_, 2 mL of 10^−3^ mol L^-1^ IAA and 5 mL of PBS (pH 6.0) ] was incubated at 25°C for 30 min. 4 mL of IAA was added to the reaction solution (2 mL) and the absorbance was measured at 530 nm 30 min later.

### RNA extraction and real-time quantitative RT-PCR analysis

Total RNA was abstracted with TaKaRa MiniBEST plant RNA extraction kit (Takara Bio Inc, Kusatsu, Shiga, Japan) according to the manufacturer’s instructions. Total RNA was reverse transcribed by PrimeScript TMRT Master Mix (Perfect Real Time) according to the manufacturer’s instructions. The gene sequence was searched in Gen Bank, and NCBI BLAST was used to compare published gene sequences from different countries and regions to find specific fragments. Then primers design were shown in Table 1. PCR cycling conditions were as follows: 3 min at 72°C followed by 40 cycles of 5 min at 95°C, 10 sec at 94°C, 30 sec at 60°C and 30 sec at 72°C with data collection at the annealing step. The expression level of the gene was calculated by 2^-ΔΔCT^. ΔCT = CT (target gene)—CT (internal reference gene). ΔΔCT = ΔCT (test group)—ΔCT (control group). The relative expression level of the gene = 2^-ΔΔCT^.

**Table 1:** Sequences of primers used for RT-PCR analysis.

Gene name	NCBI accession number	primer	5′-3′ primer sequence	Size(bp)
*APX*	NM_001250856.1	F	5’-ACAAACCCGAGCCACCA-3’	17
		R	5’-ACCACCAGAGAGAGGGCAACAC -3’	19
*MAHAR*	AB442087.1	F	5’-TGGAGTGGCAGCAGGATATG-3’	20
		R	5’-GAGGATCAATTCTATCCCTTTCTCTTC-3’	27
*DHAR*	NM_001250000.1	F	5’-TCCAGAATACCAAGGCTGAAGAA -3’	23
		R	5’- CTACCAAGACACAACACGATTACA-3’	25
*GR*	L11632.1	F	5’-TGATGAGGCTTTGAGTTTAGAGGAG-3’	25
		R	5’-AACTTTGGCACCCATACCATTC -3’	22
*actin*	DQ115883	F	5’- CACTACACCGTTGGAAAGGAAA-3’	22
		R	5’-CAAAAGGAGGGAGCCGAGA -3’	19

### Statistical analysis

Where indicated, results were expressed as mean values ± SE from at least three independent experiments. Data collected were subjected to analysis of variance (ANOVA), and statistical divergence among treatments was analyzed through Duncan’s multiple range test (*P* < 0.05). All statistical analysis was carried out using the statistical package for social science for windows (version13.00; SPSS, Inc., Chicago, IL, United States).

## Results

### Effects of hydrogen on adventitious rooting in cucumber under Cd stress

**Fig 1:**
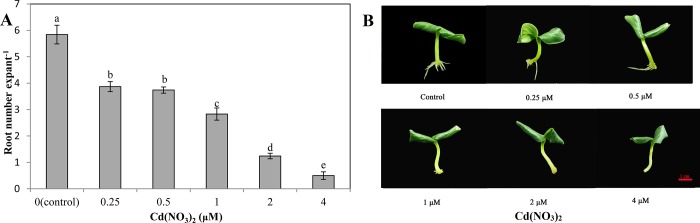
Effects of different concentrations of Cd(NO_3_)_2_ on the adventitious rooting in cucumber explants. The primary roots were removed from hypocotyls of 5-day-old cucumber seedlings. Explants were incubated for 5 days with distilled water (control) or different concentrations of Cd(NO_3_)_2_ (0.25, 0.5, 1, 2 and 4 μM). Adventitious root numbers (**A**) were expressed as mean ± SE. Bars not sharing the same letters were significantly different (*P*<0.05). Photos (**B**) were taken 5 days after the treatment, bar = 1cm.

As shown in Fig 1, compared with control (distilled water), Cd(NO_3_)_2_ treatments resulted in a significant decrease in adventitious root number. The number of adventitious roots in cucumber explants was gradually decreased as the concentration of Cd(NO_3_)_2_ increased (0.25–4 μM). Compared with control, the number of roots in 0.25 and 0.5 μM Cd(NO_3_)_2_ treatments decreased by 33.73% and 35.96%, respectively. The number of roots in 1 μM Cd(NO_3_)_2_ treatment was reduced to 48.4% of the control. Compared with control explants, the root number in explants treated with 2 and 4 μM Cd(NO_3_)_2_ decreased by 78.77% and 91.44%, respectively. These results indicated that treatments with 0.25–0.5, 1, and 2–4 μM Cd(NO_3_)_2_ could be termed as mild, moderate and severe Cd stress, respectively. As 1 μM Cd(NO_3_)_2_ induced moderate stress, the concentration was used for the next experiments.

**Fig 2:**
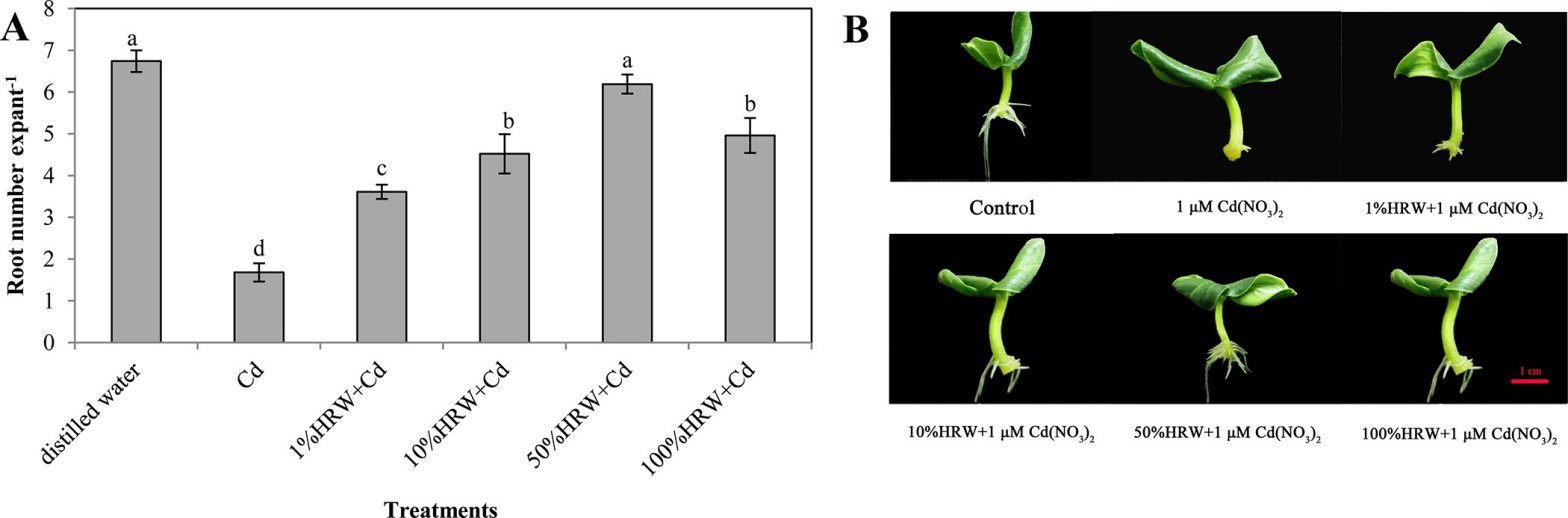
Effects of different concentrations of HRW on the adventitious rooting in cucumber explants under Cd stress. The primary roots were removed from hypocotyls of 5-day-old cucumber seedlings. Explants were incubated for 5d with distilled water (control) or different concentrations of HRW and Cd(NO_3_)_2_ co-treatment (1μM Cd(NO_3_)_2_,1% HRW+1μM Cd(NO_3_)_2_, 10% HRW+1μM Cd(NO_3_)_2_, 50%HRW+1μM Cd(NO_3_)_2_ and 100%HRW+1μM Cd(NO_3_)_2_). Adventitious root numbers (**A**) were expressed as mean ± SE. Bars not sharing the same letters were significantly different (*P*<0.05). Photos (**B**) were taken 5 days after the treatment, bar = 1cm.

Different concentrations of HRW (1%, 10%, 50%, and 100%) significantly increased the number of adventitious roots in cucumber explants under Cd stress (Fig 2). Moreover, treatment with 50% HRW resulted in a maximum remission effect almost reaching the level of the control. Therefore, the optimum concentration of HRW (50%) was used for the next experiments.

### Effects of HRW on membrane lipid peroxidation during adventitious root development in cucumber under Cd stress

**Fig 3:**
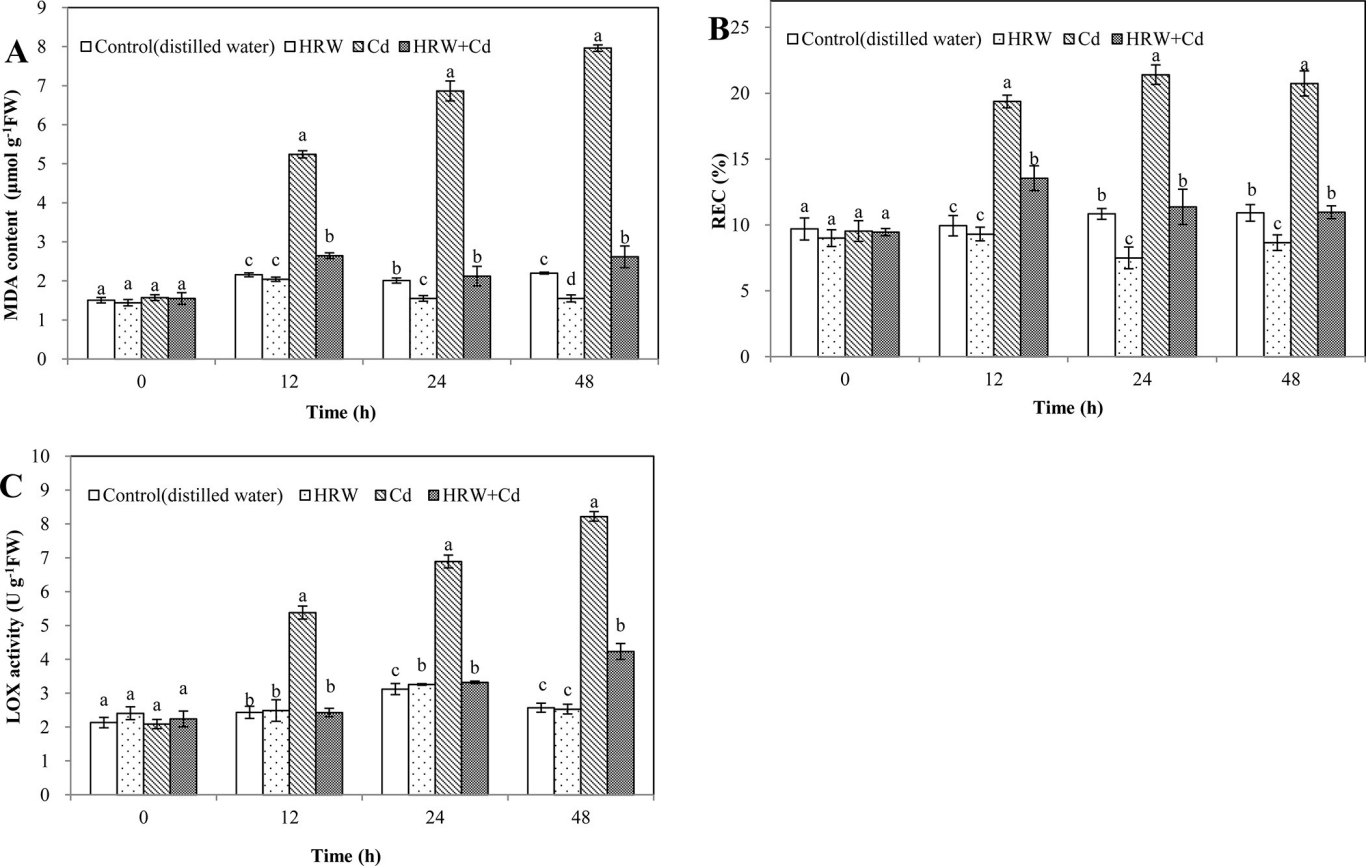
Effects of HRW on malondialdehyde (MDA) content, relative electrical conductivity (REC) and lipoxygenase (LOX) activity during adventitious root development in cucumber explants under Cd stress. The primary roots were removed from hypocotyls of 5-day-old cucumber seedlings. Explants were incubated for 2 d with distilled water (control), 50%HRW, 1μM Cd(NO_3_)_2_, 50%HRW+1μM Cd(NO_3_)_2_. MDA content (**A**), relative conductivity (**B**) and LOX activity (**C**) were measured at 0, 12, 24 and 48 h and expressed as mean ± SE, respectively. Bars not sharing the same letters were significantly different (*P*<0.05).

Compared with control, HRW treatment caused 22.87% and 29.43% decreases in MDA content at 24 and 48 h. Compared with control at 12, 24 and 48 h, Cd treatment resulted in a sharp increase in MDA content (Fig 3A). However, HRW + Cd treatment significantly decreased MDA content by 49.49%, 69.06% and 67.14% compared with Cd treatment, respectively.

At 24 and 48 h, HRW treatment significantly decreased REC by 30.76% and 20.73% compared with control, respectively. However, Cd treatment significantly increased REC by 94.84%, 97.54% and 90.02% compared with control at 12, 24, and 48 h, respectively. REC in HRW + Cd treatment was 30.05%, 46.89% and 47.16% lower than that in Cd treatment, respectively (Fig 3B).

LOX activity of HRW treatment was higher than that of control at 24 h (Fig 3C). At 12, 24 and 48 h, compared with control, Cd treatment significantly increased LOX activity. However, compared with Cd treatment, HRW + Cd treatment significantly decreased LOX activity by 54.83%, 51.81% and 72.87%, respectively. Thus it was suggested that H_2_ could alleviate membrane lipid peroxidation in Cd-treated cucumber explants and considerably enhanced adventitious root development.

### Effects of HRW on reactive oxygen species during adventitious root development in cucumber under Cd stress

**Fig 4:**
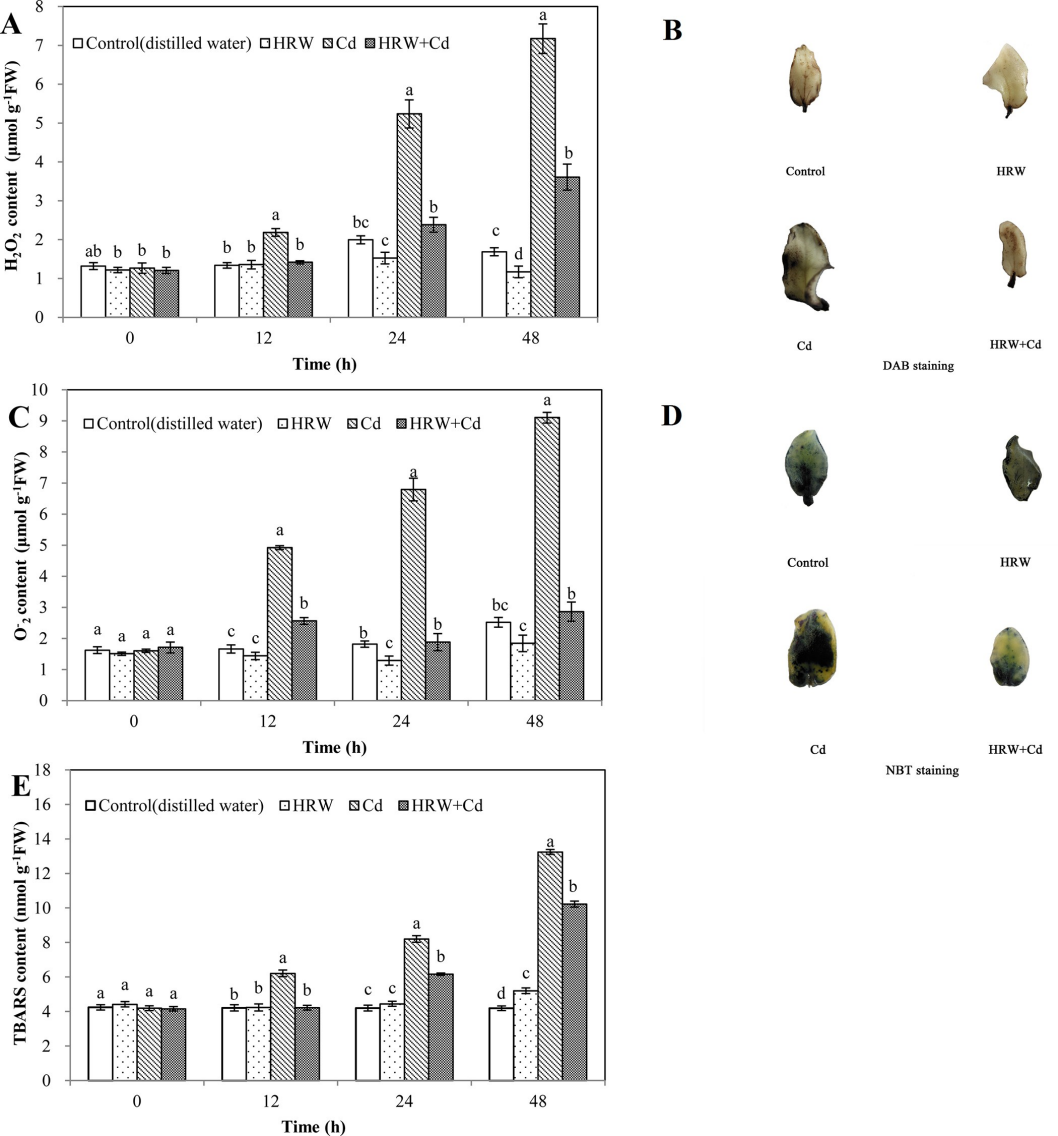
Effects of HRW on the content of H_2_O_2_, O_2_^-^ and TBARS during adventitious roots development in cucumber under Cd stress. The primary roots were removed from hypocotyls of 5-day-old cucumber seedlings. Explants were incubated for 2 d with distilled water (control), 50%HRW, 1μM Cd(NO_3_)_2_, 50%HRW + 1μM Cd(NO_3_)_2_. Photos of DAB and NBT staining were taken 24 h after the treatment (**B**, **D**).The content of H_2_O_2_ (**A**)_,_ O_2_^-^ (**C**) and TBARS (**E**) were measured at 0, 12, 24 and 48h and expressed as mean ± SE, respectively. Bars not sharing the same letters were significantly different (*P*<0.05).

As shown in Fig 4A, HRW treatment resulted in 23.58% and 30.70% decreases in H_2_O_2_ content compared with control at 24 and 48 h, respectively. H_2_O_2_ content of Cd treatment gradually increased with time. Compared with Cd treatment at 12, 24 and 48h, HRW + Cd treatment significantly caused 35.16%, 54.58% and 49.65% decreases in H_2_O_2_ content, respectively.

Under the catalysis of peroxidase, DAB and hydrogen peroxide can generate brown compounds, which can locate hydrogen peroxide in tissues (Fig 4B). Compared with control, Cd treatment resulted in more dark brown spots on the left side of the leaves. Compared with Cd treatment, HRW + Cd treatment produced a sharp decrease in the number of dark brown spots and made the color more lighter (Fig 4B).

At 24 and 48 h, O_2_^-^ content of HRW treatment was 29.13% and 27.01% lower than that of control, respectively. However, when compared with Cd treatment at 12, 24 and 48 h, HRW + Cd treatment resulted in significant decrease of 47.97%, 72.31% and 68.54% in O_2_^-^ content, respectively, almost reaching the control level (Fig 4C).

NBT reacts with O_2_^-^ under light and a dark blue precipitate can form, which can be used for O_2_^-^ tissue staining (Fig 4D). When compared with control, Cd treatment resulted in appearing blue-black for leaves, while HRW + Cd treatment made the blue-black lighter and produced a decrease in the number of spots (Fig 4D).

As shown in Fig 4E, TBARS content of HRW treatment was significantly higher 24.23% than that of control at 48 h. TBARS content of Cd treatment from 12 to 48 h was significantly higher than that of control. Compared with Cd treatment from 12 to 48 h, HRW + Cd treatment caused 32.06%, 24.97% and 22.81% decreases in TBARS content, respectively (Fig 4E). Above results clearly suggested that H_2_ could alleviate oxidation damage caused by Cd toxicity to promote adventitious rooting in cucumber.

### Effects of HRW on ascorbate-glutathione cycle during adventitious root development in cucumber under Cd stress

**Fig 5:**
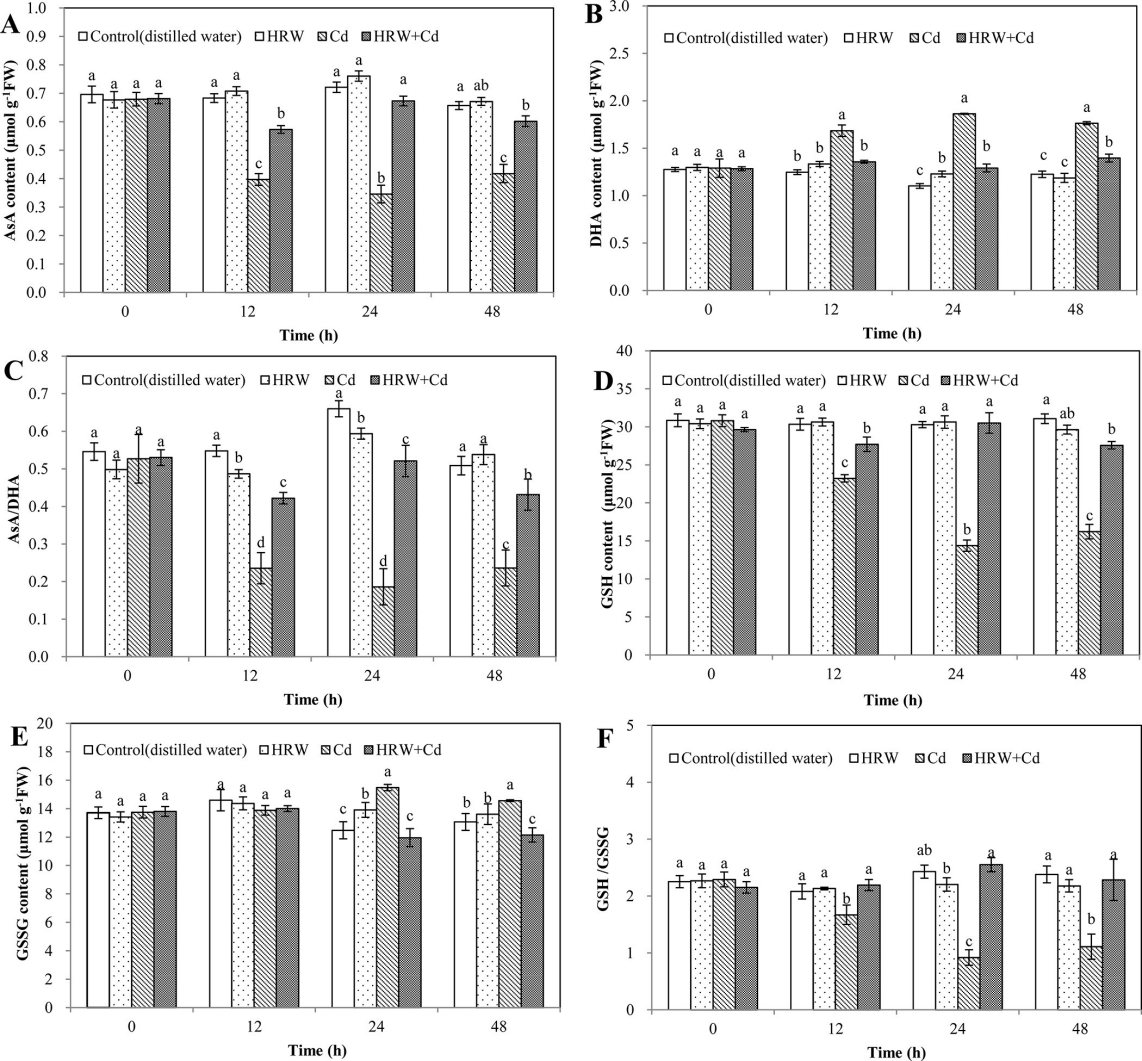
Effects of HRW on AsA content, DHA content, AsA/DHA, GSH content, GSSG content and GSH/GSSG during adventitious roots development in cucumber under Cd stress. The primary roots were removed from hypocotyls of 5-day-old cucumber seedlings. Explants were incubated for 2 d with distilled water (control), 50%HRW, 1μM Cd(NO_3_)_2_, 50%HRW+1μM Cd(NO_3_)_2_. AsA/DHA (**C**), GSH/GSSG (**F**) and the content of AsA (**A**), DHA (**B**), GSH (**D**) and GSSG (**E**) were measured at 0, 12, 24 and 48h and expressed as mean ± SE, respectively. Bars not sharing the same letters were significantly different (*P*<0.05).

Compared with control at 12, 24 and 48 h, Cd treatment produced a significant decrease in AsA content (Fig 5A). However, compared with Cd treatment, HRW + Cd treatment significantly increased AsA content by 44.33%, 94.51% and 44.02%, respectively (Fig 5A).

As shown in Fig 5B, Cd treatment caused 35.15%, 69.01% and 44.00% increases in DHA content compared with control at 12, 24 and 48 h, respectively. At 24 h, HRW treatment produced an 11.51% increase in DHA content compared with the control. At 12, 24 and 48 h, HRW + Cd treatment significantly decreased DHA content by 19.48%, 30.69% and 20.88% compared with Cd treatment, respectively (Fig 5B).

At 12 and 24 h, HRW treatment significantly decreased AsA/DHA ratio by 11.19% and 10.06% compared with control, respectively (Fig 5C). However, AsA/DHA ratio of HRW treatment was significantly higher 5.76% than that of control at 48 h. At 12, 24, and 48 h, AsA/DHA ratio of Cd treatment was significantly lower than that of control. At 12, 24 and 48 h, HRW + Cd treatment significantly increased AsA/DHA ratio by 79.19%, 180.22% and 82.75% compared with Cd treatment, respectively (Fig 5C).

At 12, 24 and 48 h, compared with the control, Cd treatment resulted in a significant reduce in GSH content. However, GSH content of HRW + Cd treatment increased by 19.32%, 112.17% and 70.06% compared with that of Cd treatment at 12, 24 and 48 h, respectively (Fig 5D).

GSSG content of HRW treatment was significantly higher 11.52% than that of control at 24 h. At 24 and 48 h, GSSG content of Cd treatment was significantly higher than that of control. Compared with Cd treatment at 24 and 48h, HRW + Cd treatment significantly decreased GSSG content by 22.76 and 16.55%, respectively (Fig 5E).

During 12 to 48 h, GSH/GSSG ratio of Cd treatment was significantly lower than that of control. Compared with Cd treatment at 12, 24 and 48 h, HRW + Cd treatment caused 31.35%, 177.34% and 105.60% increases in GSH/GSSG ratio, respectively (Fig 5F). Thus, H_2_ could maintain ascorbate and glutathione homeostasis, which enhanced the antioxidant capacity and promoted cucumber adventitious rooting.

**Fig 6:**
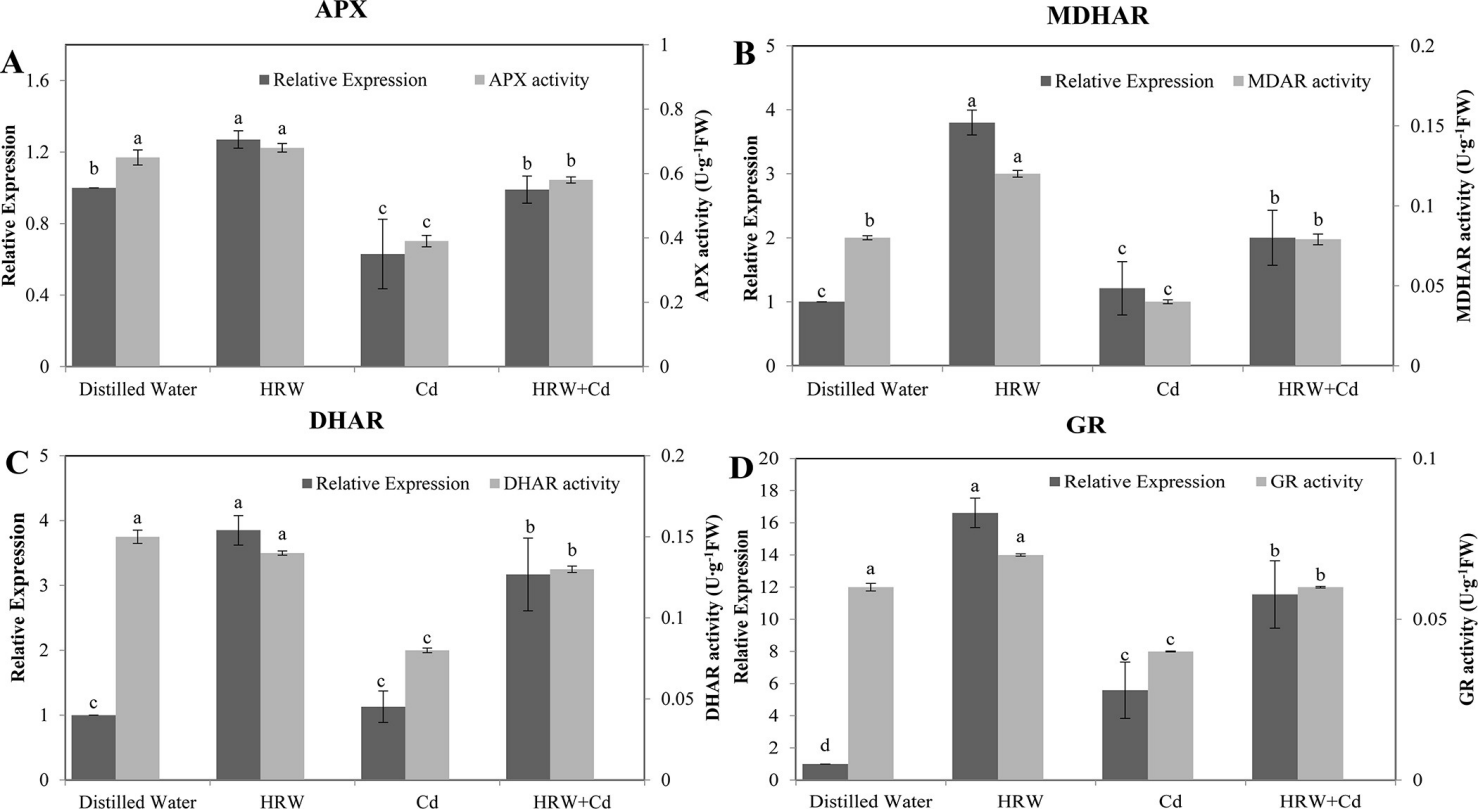
Effects of HRW on Ascorbate-glutathione cycle related enzyme activity and gene expression during adventitious root development in cucumber under Cd stress. The primary roots were removed from hypocotyls of 5-day-old cucumber seedlings. Explants were incubated for 2 d with distilled water (control), 50%HRW, 1μM Cd(NO_3_)_2_, 50%HRW+1μM Cd(NO_3_)_2_. The relative enzyme expression and activity of APX (**A**), MDHAR (**B**), DHAR (**C**) and GR (**D**) were expressed as mean ± SE, respectively. Bars not sharing the same letters were significantly different (*P*<0.05).

The change of relative expression level of APX, MDHAR and GR with time was consistent with the change of corresponding enzyme activity (Fig 6A, 6B, and 6D). Relative expression of APX in HRW treatment was significantly higher than that of control (Fig 6A). Compared with control, Cd treatment significantly decreased APX relative expression by 37% and APX activity by 40%, respectively. However, compared with Cd treatment, HRW + Cd treatment produced a 57.14% increase in APX relative expression and a 48.72% increase in APX activity, respectively (Fig 6A).

HRW treatment resulted in a 280% increase in MDHAR relative expression and a 50% increase in MDHAR activity compared with control, respectively (Fig 6B). MDHAR activity of Cd treatment was significantly lower than that of control. HRW + Cd treatment resulted in a 65.29% increase in MDHAR relative expression and a 100% increase in MDHAR activity compared with Cd treatment, respectively.

HRW treatment significantly increased DHAR relative expression by 285% compared with control (Fig 6C). Compared with control, Cd treatment significantly decreased DHAR activity by 42.86%. HRW + Cd treatment had a 180.53% increase in DHAR relative expression and a 62.5% increase in DHAR activity compared with Cd treatment, respectively.

GR relative expression of HRW treatment was significantly higher 1562% than that of control. Cd treatment increased GR relative expression by 459% and caused a 33.33% decrease in GR activity compared with control (Fig 6D). HRW + Cd treatment resulted in a 106.62% increase in GR relative expression and a 50% increase in GR activity compared with Cd treatment (Fig 6D). Thus, H_2_ promoted adventitious root development under Cd stress by regulating ascorbate-glutathione cycle-related enzyme activity and gene expression.

### Effects of HRW on osmotic adjustment substances during adventitious root development in cucumber under Cd stress

**Fig 7:**
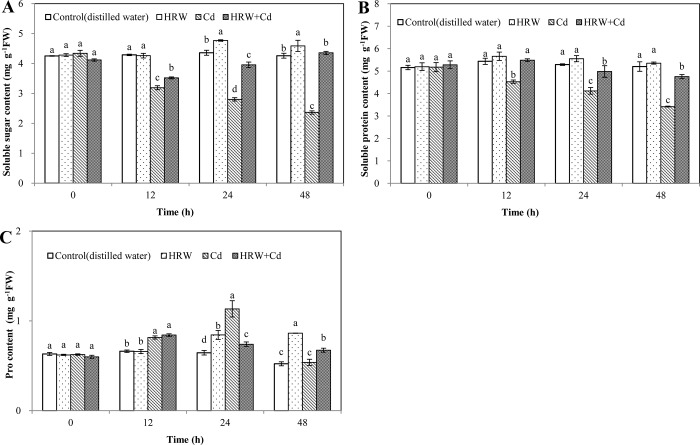
Effects of HRW on the content of soluble sugar, soluble protein and Pro during adventitious root development in cucumber under Cd stress. The primary roots were removed from hypocotyls of 5-day-old cucumber seedlings. Explants were incubated for 2 d with distilled water (control), 50%HRW, 1μM Cd(NO_3_)_2_, 50%HRW+1μM Cd(NO_3_)_2_. The content of soluble sugar (**A**), soluble protein (**B**)and pro (**C**) were measured at 0, 12, 24 and 48h and expressed as mean ± SE, respectively. Bars not sharing the same letters were significantly different (*P*<0.05).

Soluble sugar content of HRW treatment was significantly higher than that of control at 24 and 48 h (Fig 7A). However, soluble sugar content of Cd treatment was significantly lower than that of control at 12, 24, and 48 h. Compared with Cd treatment at 12, 24 and 48 h, HRW + Cd treatment significantly increased soluble sugar content by 10.34%, 41.43% and 84.75%, respectively.

Compared with control, HRW treatment resulted in an increase in soluble protein content. Soluble protein content of Cd treatment was 16.73%, 22.12% and 34.23% lower than that of control at 12, 24 and 48 h, respectively. However, compared with Cd treatment, HRW + Cd treatment significantly increased soluble protein content (Fig 7B).

HRW treatment caused 30.70% and 65.33% increases in pro content compared with control at 24 and 48 h, respectively (Fig 7C). However, Cd treatment increased proline content by 23.11% and 75.81% compared with control at 12 and 24 h, respectively. Compared with Cd treatment, HRW + Cd treatment resulted in a 34.52% decrease at 24 h and a 24.07% increase in pro content at 48 h, respectively (Fig 7C). Therefore, H_2_ could increase osmotic adjustment substances content to alleviate Cd toxicity during rooting.

### Effects of HRW on rooting-related enzymes during adventitious root development in cucumber under Cd stress

**Fig 8:**
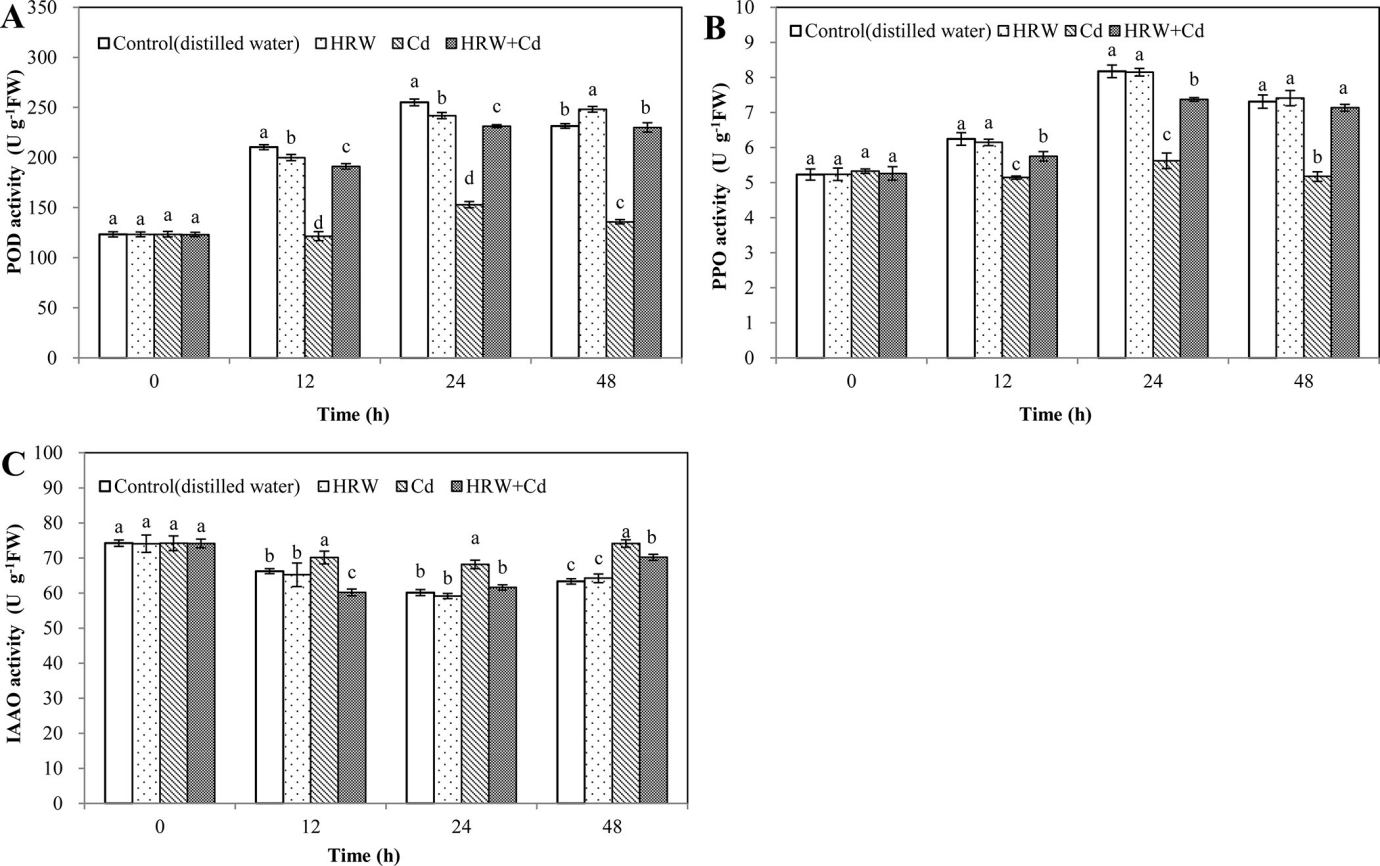
Effects of HRW on the activity of POD, PPO and IAAO during adventitious root development in cucumber under Cd stress. The primary roots were removed from hypocotyls of 5-day-old cucumber seedlings. Explants were incubated for 2 d with distilled water (control), 50%HRW, 1μM Cd(NO_3_)_2_, 50%HRW + 1μM Cd(NO_3_)_2_. The activity of POD (**A**), PPO (**B**) and IAAO (**C**) were measured at 0, 12, 24 and 48h and expressed as mean ± SE, respectively. Bars not sharing the same letters were significantly different (*P*<0.05).

As shown, at 48 h, POD activity of HRW treatment was significant higher than that of control. At 12, 24 and 48 h, Cd treatment significantly resulted in 42.23%, 40.01% and 41.30% decrease in POD activity compared with control, respectively. However, compared with Cd treatment, HRW + Cd treatment significantly increased POD activity by 57.34%, 51.12% and 69.27%, respectively (Fig 8A).

At 12, 24 and 48 h, Cd treatment significantly decreased PPO activity by 17.66%, 31.23% and 29.26% compared with control, respectively. At 12, 24 and 48 h, PPO activity of HRW + Cd treatment was 11.78%, 31.11% and 37.92% higher than that of Cd treatment, respectively (Fig 8B).

At 12, 24, and 48 h, Cd treatment significantly caused 5.87%, 13.36% and 17.06% increases in IAAO activity compared with control, respectively. However, compared with Cd treatment, HRW + Cd treatment significantly decreased IAAO activity (Fig 8C). These data indicated that H_2_ could regulate activities of rooting-related enzymes and alleviate oxidation damage caused by Cd toxicity to promote explants adventitious rooting.

## Discussion

H_2_ has been shown to be involved in various physiological processes of plants, from developmental processes to plant responses in abiotic stresses [9]. For example, HRW alleviated Cd toxicity in *Medicago sativa* seedlings [27]. Recently, it has been reported that H_2_ had a positive effect on adventitious root development in cucumber explants under stress-free conditions [17, 34]. Our results clearly demonstrated that HRW promoted adventitious rooting in cucumber under Cd stress, with a maximal biological response at 50% HRW (Fig 2A and 2B). Wu et al [35] also found that H_2_ promoted root growth in Chinese cabbage under Cd stress. Several aspects may account for the positive effects of HRW on promoting adventitious rooting in cucumber under Cd stress and are discussed in the following.

MDA content, REC and LOX activity were considered as effective indicators of the cell membrane lipid peroxidation during response to various environmental stresses. In this study, we provided evidence of the beneficial effect of H_2_ on the development of cucumber adventitious roots upon Cd stress. This conclusion was confirmed by evaluating its alleviating performance on the cell membrane lipid peroxidation caused by Cd toxicity, including the excessive increase of MDA content, REC and LOX activity (Fig 3). Our results suggested that Cd stress considerably increased plasma membrane permeability and destroyed the membrane lipid stability, more importantly, H_2_ could alleviate membrane lipid peroxidation in Cd-treated cucumber explants. Similar to our results, Nahar et al [36] demonstrated that a large increase in LOX activity and MDA content was involved in membrane lipid peroxidation in *Vigna radiata* L. seedlings under Cd stress. Additionally, Ahmad et al [37] also found that REC increased in mustard (*Brassica juncea*) leaves exposed to Cd stress. Xu et al [38] also observed that HRW could reduce the accumulation of MDA in rice under salt stress. LOX activity in the roots of *M*. *sativa* was also decreased by HRW treatment under Cd stress [27]. Meanwhile, Chen et al [19] reported that HRW significantly decreased REC in cucumber leaves under heat stress. Moreover, overproduction of ROS and subsequent oxidative stress is the mechanisms of phytotoxicity of Cd. O^−^_2_, H_2_O_2_ and TBARS content could reflect the oxidative damage degree to membrane lipids. Our further experiments revealed that HRW was able to regulate Cd-triggered oxidative stress during cucumber adventitious rooting. For example, the Cd-induced increase of TBARS, H_2_O_2_ and O^-^_2_ content was reduced by HRW (Fig 4A, 4C and 4E). This conclusion was confirmed by histochemical staining of ROS (Fig 4B and 4D). It was also reported that the content of TBARS, H_2_O_2_ and O^-^_2_ was increased in the rice seedlings under Cd, lead (Pb) or Cd + Pb stress [39]. Similarly, the positive roles of HRW in reducing the content of TBARS, H_2_O_2_ and O^-^_2_ have been reported in *M*. *sativa* plants under paraquat stress [40]. Therefore, we provided evidence that H_2_ could alleviate oxidation damage caused by Cd toxicity to protect the stability of cell membrane and promote the adventitious rooting in cucumber.

Obviously, avoiding oxidative stress and reestablishing redox homeostasis are of vital importance in alleviation of metal stress [39]. Therefore, reestablishment of redox homeostasis is another possible explanation of HRW responses in the alleviation of Cd toxicity during promoting adventitious rooting. There are two types of protection systems in plants: one is a non-enzymatic protection system such as AsA, GSH, DHA and GSSG; another is an enzymatic protection system including APX, GR, MDHAR and DHAR. Here, the decreases of AsA/DHA and GSH/GSSG, which may cause oxidative damage in plants, were observed in Cd-treated cucumber explants, suggesting the cellular redox imbalance upon Cd exposure (Fig 5). An increase in GSSG and a decrease in GSH/GSSG ratio in rapeseed seedlings reflected the oxidative stress induced by Cd [41]. Srivastava et al [39] also observed that Cd, Pb as well as Cd+Pb treatments increased DHA level and declined AsA level as well as AsA/DHA ratio in rice seedlings. In our study, we also found that the Cd-induced decreases in AsA/DHA and GSH/GSSG ratio were reversed by HRW, indicating that H_2_ alleviated oxidative stress caused by Cd, which was agreement with the beneficial effects on the alleviation of oxidative stress, and the decreased ROS distribution discussed above. Similarly, Cui et al [42] found that HRW significantly increased reduced/oxidized AsA and reduced/oxidized GSH ratios in alfalfa seedlings upon HgCl_2_. Thus, H_2_ may help plants alleviating heavy metal stress by increasing AsA/DHA and GSH/GSSG ratios and consequently reestablish cellular redox balance. It was previously confirmed that anti-oxidant enzymes in plants including APX and other antioxidant enzymes counteracted abiotic stress-induced ROS accumulation. Comparatively, the changes in activities of APX, DHAR, MDHAR, and GR (Fig 6), responsible for GSH, GSSG, AsA and DHA metabolism, were consistent with the changes in GSH/GSSG and AsA/DHA ratios induced by HRW under Cd stress. Under heat stress, APX and GR activities may be reduced, which caused more severe membrane overoxidation [43]. MDHAR, DHAR and GR activities decreased in *Brassica napus* seedlings under Cd stress [44]. Similar to H_2_, nitric oxide reduced oxidative damage and increased AsA and GSH content and activities of APX, DHAR, MDHAR and GR in mung bean under Cd stress [36]. Upon heavy metals stress, however, the possibility of the increased ROS-triggered expression of genes for antioxidant enzymes was also shown [45]. H_2_ can readily permeate the cell membrane and thereby regulating gene expression of these enzymes. Our further experiments suggested that HRW could enhance the gene expression level of Ascorbate-glutathione cycle related enzymes, indicating that H_2_ may help maintain ascorbate and glutathione homeostasis to reestablish redox balance and alleviate peroxidation damage in Cd-treated cucumber explants. Above results suggested that H_2_ had a positive effect on enhancing the antioxidant capacity, inhibiting the production and accumulation of ROS in Cd-treated cucumber explants and promoting cucumber adventitious rooting.

Soluble sugars and proline can regulate osmotic potential to improve membrane permeability, maintain water homeostasis and enhance antioxidant defense mechanisms under abiotic stresses. Our results suggested that Cd stress destroyed defense mechanisms and reduced osmoprotectants such as pro, soluble proteins and sugars, while HRW promoted the accumulation of osmoprotectants under Cd stress (Fig 7). In consistent with our results, Chen et al [19] also showed that HRW significantly increased the levels of pro and soluble sugars in cucumber leaves under high temperature stress. Under abiotic stresses, HRW-promoted adventitious rooting may be due to the balance of ROS and the increase in osmoprotectants. Besides, it has been reported that IAAO, PPO and POD activities could promote adventitious root formation [46]. Lower IAAO activity was useful for rooting in easy-to-root cultivar as its lower ability to degrade IAA [47]. Previous study has suggested that PPO activity was increased greatly during root formation [46]. POD activity was reduced under drought stress, while HRW increased the activity of POD during cucumber adventitious rooting under drought stress [23]. Kováčik et al [48] reported that 120 mM nickel decreased PPO activity in roots of *M*. *chamomilla*. HRW increased PPO and POD activities during adventitious rooting [49]. Zhu et al [33] also found that HRW significantly reduced IAAO activity in the cucumber explants. Cd stress diminished activities of PPO and POD and increased IAAO activity, while HRW increased POD and PPO activities and repressed IAAO activity, resulting in enhancement of adventitious rooting under Cd stress (Fig 8). Therefore, these results indicated that H_2_ could regulate activities of rooting-related enzymes, increase osmotic adjustment substances content, resulting in maintaining cell membrane integrity during rooting.

Taken together, H_2_ had a positive effect in promoting explants adventitious rooting under Cd stress. During that process, H_2_ alleviated membrane lipid peroxidation, inhibited the production and accumulation of ROS by regulating glutathione antioxidant defense system, and increased osmotic adjustment substances content. Additionally, H_2_ could regulate activities of rooting-related enzymes. Therefore, this study indicated that the positive role of H_2_ in adventitious rooting under heavy metal stress, which could be explored in agricultural production activities. However, more genetic and molecular methods are needed to further demonstrate the detailed molecular mechanisms during that process.

## Supporting information

S1 Table Effects of hydrogen on adventitious rooting. (XLSX)

S2 Table Membrane lipid peroxidation date. (XLSX)

S3 Table Reactive oxygen species date. (XLSX)

S4 Table Ascorbate-glutathione cycle date. (XLSX)

S5 Table Osmotic adjustment substances date. (XLSX)

S6 Table Rooting-related enzymes date. (XLSX)
